# Injectable hydrogel loaded with bilayer microspheres to inhibit angiogenesis and promote cartilage regeneration for repairing growth plate injury

**DOI:** 10.3389/fbioe.2023.1181580

**Published:** 2023-05-18

**Authors:** Lei Qiang, Minjie Fan, Yiwei Wang, Yihao Liu, Hanjie Zhuang, Ruoyi Guo, Hao Huang, Yulong Ben, Dalin Wang, Xiaoling Wu, Jinwu Wang, Jie Weng, Pengfei Zheng

**Affiliations:** ^1^ Department of Orthopaedic Surgery, Children’s Hospital of Nanjing Medical University, Nanjing, Jiangsu, China; ^2^ Key Laboratory of Advanced Technologies of Materials (MOE), School of Materials Science and Engineering, Southwest Jiaotong University, Chengdu, Sichuan, China; ^3^ Shanghai Key Laboratory of Orthopaedic Implant, Department of Orthopaedic Surgery, Shanghai Ninth People’s Hospital, Shanghai Jiao Tong University School of Medicine, Shanghai, China; ^4^ Department of Orthopaedic Surgery, Nanjing First Hospital, Nanjing, Jiangsu, China; ^5^ School of Biomedical Engineering and Informatics, Nanjing Medical University, Nanjing, Jiangsu, China

**Keywords:** growth plate, bilayer microspheres, anti-angiogenesis, chondrogenic differentiation, hydrogel, tissue engineering

## Abstract

**Introduction:** The repair and regeneration of growth plate injuries using tissue engineering techniques remains a challenge due to large bone bridge formation and low chondrogenic efficiency.

**Methods:** In this study, a bilayer drug-loaded microspheres was developed that contains the vascular endothelial growth factor (VEGF) inhibitor, Bevacizumab, on the outer layer and insulin-like growth factor-1 (IGF-1), a cartilage repair factor, on the inner layer. The microspheres were then combined with bone marrow mesenchymal stem cells (BMSCs) in the gelatin methacryloyl (GelMA) hydrogel to create a composite hydrogel with good injectability and biocompatibility.

**Results:** The in vitro drug-release profile of bilayer microspheres showed a sequential release, with Bevacizumab released first followed by IGF-1. And this hydrogel simultaneously inhibited angiogenesis and promoted cartilage regeneration. Finally, in vivo studies indicated that the composite hydrogel reduced bone bridge formation and improved cartilage regeneration in the rabbit model of proximal tibial growth plate injury.

**Conclusion:** This bilayer microsphere-based composite hydrogel with sequential controlled release of Bevacizumab and IGF-1 has promising potential for growth plate injury repair.

## 1 Introduction

Growth plate is the critical cartilage area at the end of long bones in children, responsible for controlling the longitudinal growth of bone (Kronenberg, 2003; Agirdil, 2020). Notably, the growth plate is avascular and devoid of nerves (Provot and Schipani, 2007; Villemure and Stokes, 2009), which limits their inherent ability to repair after injury. In such cases, the cartilage may be replaced by bone tissue, leading to the formation of bone bridge. The bone bridge could impede bone growth and result in significant complications, including length discrepancy and angular deformity, which could negatively impacts on the physical and mental wellbeing of affected children (McCarty et al., 2010; Cepela et al., 2016; Shaw et al., 2018). The current clinical treatment mainly involves osteotomy combined with filling the corresponding materials such as fat and bone cement. However, this approach has limited efficacy and a low success rate (Foster et al., 2000; Hasler and Foster, 2002). Thus, there is an urgent pressing need for new and more effective treatments for injuries to growth plates.

Recently, advancements in tissue engineering have enabled significant progress in the repair of injured growth plates. This is achieved by incorporating a combination of bone marrow mesenchymal stem cells (BMSCs) or chondrocytes and growth factors into biologically active scaffolds, aimed at constructing the growth-plate-regenerative cartilage that can replace the injured tissue and perform its original biological function (Xian and Foster, 2006; Shukrimi et al., 2013; Wang et al., 2021). For instance, Erickson et al. demonstrated the use of chitosan-genipin microgels loaded with stromal-cell derived factor-1α (SDF-1α) or transforming growth factor β3 (TGF-β3) (Erickson et al., 2021a). Li et al. showed the feasibility of repairing growth plate cartilage by loading BMSCs on extracellular matrix scaffolds (Li et al., 2017). Erickson et al. explored the use of alginate-chitosan hydrogels loaded with VEGF antibodies (Erickson et al., 2021b). Despite these promising results, these studies reported the challenges of achieving efficient chondrogenic differentiation and avoiding the formation of large bone bridges (Azarpira et al., 2015; Sundararaj et al., 2015; Li et al., 2017; Erickson et al., 2021a; Erickson et al., 2021b). Our hypothesis is that these challenges can be overcome by implementing a sequential approach that targets both the prevention of bone bridge formation and the promotion of chondrogenic differentiation in the early stages of injury. By targeting a series of cellular and molecular events following injury (Chung and Xian, 2014), and blocking or even reversing these pathological changes, it may be possible to achieve more effective regeneration and repair of growth plate cartilage.

Over the past two decades, extensive research using various animal models has been performed to gain a better understanding to the underlying pathophysiology of the bone bridge formation following injury (Zhou et al., 2004; Chung et al., 2009; Chung et al., 2011; Xie et al., 2014). The research has identified four different phases of injury repair: the inflammatory phase, the fibrogenic phase, the osteogenic phase, and the remodeling phase (Chung and Xian, 2014). The initial phase, lasting 1–3 days post-injury, is characterized by the infiltration of inflammatory cells and the upregulation of cytokines and mediators at the injury site. This constitutes the inflammatory phase of growth plate injury repair. In the subsequent fibrogenic phase, which lasts 3–7 days, osteoprogenitor cells infiltrate the injury site and secrete pro-angiogenic factors. This promotes the invasion of new blood vessels, a critical step in the formation of the bone bridge (Qiu et al., 2020; Chen et al., 2021). The fibrogenic phase is therefore a crucial stage in the repair process. One of the critical pro-angiogenic factors involved in the formation of new blood vessels is vascular endothelial growth factor (VEGF), which has been shown to play a key role in bone bridge formation (Hu and Olsen, 2016; Erickson et al., 2021b; Ye et al., 2021). To prevent bone bridge formation, it is therefore essential to inhibit the action of VEGF. The osteogenic phase commences around day 7 post-injury, as osteoprogenitor cells differentiate into osteoblasts and bony trabeculae begin to form. Finally, on day 14, bone remodeling is observed. Thus, in order to interrupt the pathological process of osteogenic differentiation and prevent bone bridge formation, it is crucial to inhibit osteogenic differentiation in the early stages of growth plate injury repair. In this study, we proposed to use the VEGF inhibitor Bevacizumab (Chase, 2008; Chung et al., 2014) to inhibit angiogenesis during the fibrogenic phase, interrupting the pathological process of osteogenic differentiation and preventing the eventual formation of bone bridge. In addition, we need to induce exogenous or endogenous stem cells to differentiate into chondrocytes in order to promote the regeneration of cartilage at the injury site. Insulin-like growth factor-1 (IGF-1), a commonly used cartilage repair factor, has the ability to promote chondrogenic differentiation, chondrocyte proliferation, and matrix synthesis (Lui et al., 2019; Cho et al., 2020). In light of the protracted nature of growth plate repair, it is imperative to maintain the optimal concentration of drugs in the affected area to ensure effective repair. To this end, we have proposed the utilization of microspheres to achieve sustained delivery of Bevacizumab and IGF-1. As a well-established system for sustained drug release, poly (lactic-co-glycolic acid) (PLGA) microspheres are capable of delivering the loaded drugs in a slow and continuous manner (Morille et al., 2016; Su et al., 2021; Chen et al., 2023).

In this study, we aim to address the long-standing challenge of growth plate injury repair through a novel approach. We propose the use of a bilayer drug-loaded PLGA microsphere system that comprises of Bevacizumab and IGF-1 to achieve sustained drug delivery to the injury site. The outer layer of the microsphere is loaded with Bevacizumab, which will be released first to inhibit early osteogenic differentiation and prevent the formation of a bone bridge. The inner layer of the microsphere contains IGF-1, which will be released slowly to promote chondrogenic differentiation and the regeneration of growth plate cartilage. Further, we encapsulate the bilayer drug-loaded PLGA microspheres in a composite hydrogel, composed of gelatin methacryloyl (GelMA) and bone marrow mesenchymal stem cells (BMSCs). This composite hydrogel can provide a favorable microenvironment for both drug delivery and cell proliferation, thus facilitating the repair and regeneration of growth plate cartilage after injury. In conclusion, this study provides a novel approach for growth plate injury repair by using a bilayer drug-loaded PLGA microsphere system combined with a composite hydrogel and the results of this study hold the potential to inform the development of a clinically viable treatment strategy for growth plate injuries.

## 2 Materials and methods

### 2.1 Materials

**FIGURE 1:**
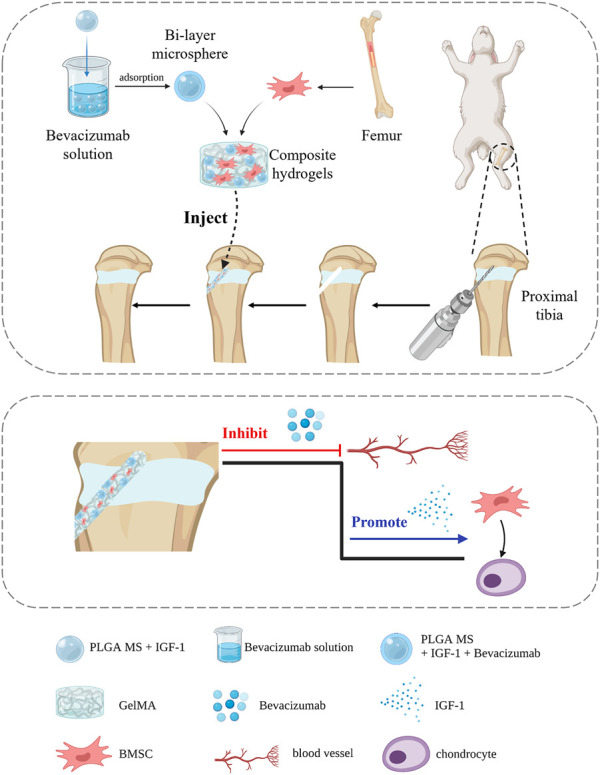
Schematic illustration of the study design.

PLGA (lactide/glycolide ratio 50:50, MW 65 kDa) and poly (vinyl alcohol) (PVA) (88%, MW 31–50 kDa) were purchased from Sigma-Aldrich (St. Louis, MO, USA). IGF-1 and Bevacizumab, Minimal Essential Medium (MEM), and phosphate-buffered solution (PBS) were obtained from Gibco (Grand Island, USA) and the RNA extraction kit was purchased from Omega Bio-tek (Georgia, USA). The cell counting kit 8 (CCK-8) was purchased from Abcam (UK); all were used as received. The study design is illustrated in Figure 1.

### 2.2 Synthesis and characterization of bilayer microspheres

In this study, microspheres were fabricated by the water-in-oil-in-water (w/o/w) double emulsion technique. Briefly, 200 mg of PLGA was dissolved in 4 mL of dichloromethane, and 500 μL of 100 μm IGF-1 solution and 150 μL of 6% gelatin were added to it. This was followed by emulsification at 10,000 rpm for 1 min by using a high-speed homogenizer. The resultant emulsion was added to 30 mL of 1% PVA solution and emulsified at 8,000 rpm for 2 min. This emulsion was added to 200 mL of 0.1% PVA solution and stirred at 1,000 rpm for 5 min, resulting in a double emulsion. Next, the emulsion was stirred in a fume hood for 4 h to evaporate dichloromethane. Finally, the solution was centrifuged to collect microspheres, and these microspheres were washed with distilled water four times and lyophilized to obtain monolayer microspheres loaded with IGF-1. Next, 100 mg of IGF-1-loaded monolayer microspheres were immersed in the PBS solution with 100 μg of Bevacizumab; after adsorption for 1 h and lyophilization, bilayer microspheres were obtained. Similarly, Bevacizumab-loaded monolayer microspheres were prepared by adding 100 mg of blank microspheres to the PBS solution containing 100 μg of Bevacizumab. The morphological characteristics of all microspheres were observed by scanning electron microscopy (SEM), and the mean size was measured. Encapsulation efficiency was calculated using the following equation: encapsulation efficiency (%) = (measured protein concentration/theoretical protein concentration) × 100 (Eswaramoorthy et al., 2012).

### 2.3 Fabrication and characterization of composite hydrogels

First, 10 g of gelatin (Sigma-Aldrich) was added to PBS at 50°C to prepare a 10% w/v homogeneous solution. Then, 10 ml of methacrylic anhydride (MA; Sigma-Aldrich) was slowly added dropwise into the gelatin solution with stirring, and the solution was allowed to react at 50°C for 3 h. Next, the resultant solution was packed into 3,500 Da dialysis bags and dialyzed in deionized water at 37°C for 5 days to remove unreacted MA and additional by-products. The dialysate was centrifuged at 3,000 rpm for 10 min, and the supernatant was lyophilized at −80°C for 6 days to obtain GelMA, which was stored at −20°C until further use.

Then, 1 g of GelMA was dissolved in 10 ml of deionized water to obtain a 100 mg/ml GelMA solution, and 0.2% (w/v) of the photoinitiator lithium phenyl-2,4,6-trimethylbenzoylphosphinate was added. Finally, BMSCs and PLGA microspheres were added at a concentration of (5×10^4^)/ml and 5 mg/ml, respectively, to form the composite hydrogel loaded with PLGA microspheres and BMSCs, and it was cross-linked under Ultraviolet (UV) light. The following groups of hydrogels were prepared: (i) GP group: GelMA + BMSCs; (ii) GPB group: GelMA + BMSCs + PLGA microspheres loaded with Bevacizumab; (iii) GPI: GelMA + BMSCs + PLGA microspheres loaded with IGF-1; (iv) GPIB group: GelMA + BMSCs + bilayer PLGA microspheres.

The injectability of composite hydrogels was analyzed by injecting the prepolymer solution carrying red pen ink with a 1 mL syringe (needle diameter = 0.5 mm). The gelatin solidification time was measured as follows: the hydrogel was injected into the glass bottle that was then gently shaken. Subsequently, the tilting angle of the glass bottle was continuously changed to observe the flow of the prepolymer solution. When the glass bottle was tilted but the solution no longer flowed, the hydrogel was considered to have been formed, and this solidification time was recorded. The viscosity and shear rate of the prepolymer solution of each group was measured by using a modular compact rheometer. Scanning electron microscopy (SEM, TESCAN) was utilized to observe the morphology of composite hydrogels. The swelling capacity of composite hydrogels was evaluated as follows (Verma et al., 2007): First, all the hydrogels were lyophilized and weighed to determine the dry weight (Md). Then, the lyophilized hydrogels were soaked in PBS at 37°C for 24 h. After removing free water, the weight was marked as Mw. Mw/Md was the swelling ratio, and [(Mw - Md)/Mw]×100% was used to calculate the equilibrium water content (EWC).

### 2.4 Mechanical characterizations and release profile

The elastic modulus was determined by using a mechanical tester (Hengyi, Shanghai, China). The samples were subjected to uniaxial compression testing at a steady strain rate of 1 mm/min and 0.1–5 Hz frequency at 25°C, and the elastic modulus was obtained by calculating the slope of the stress–strain curve for 0%–10% strain. Moreover, the original height of the sample before compression was recorded as H_0_, and after the compression test, the final height of the sample was recorded as H_r_. The ratio H_r_/H_0_ was used to measure the elasticity of the sample. The following rheological measurements were conducted at 37°C on a Haake Mars Modular Advanced Rheometer (Thermo Fisher Scientific, USA). At a fixed strain of 5%, a frequency sweep test was performed from 0.1 to 10 Hz to obtain the storage modulus (G′) and loss modulus (G″).

Next, 100 mg of the composite hydrogel from each group was immersed in 50 ml of PBS, and the drug release was analyzed in a shaker at 37°C. At predetermined time points, i.e., days 1, 3, 5, 7, 9, 11, 13, 15, 17, 19, and 21, 2 ml of the supernatant was collected and replaced with fresh PBS. The absorbance of the collected samples was measured by using a fluorescence spectrometer to evaluate the drug concentration and plot the *in vitro* drug release curve.

### 2.5 Cell viability, proliferation and adhesion

In a 96-well plate, BMSCs were seeded into the hydrogel of each group (5×10^3^/well). The culture medium was removed on days 1, 4, and 7, and washed twice with PBS. Then, 90 μL of the medium and 10 μL of CCK-8 reagent were added to each well, followed by incubation in an incubator for 1 h in the dark. The absorbance values were detected at 450 nm using the enzyme marker. To assess the cell viability on the hydrogels, BMSCs with a density of 5×10^3^ were seeded into a 12-well plate and co-incubated with hydrogels of each group for 24 h. Further, 5 μL of calcein-AM and 20 μL of PI were added to 10 mL of PBS to prepare the Live/Dead solution. Subsequently, 200 μL of the Live/Dead solution was added to each well, and incubated for 30 min at 37°C in the dark. After the solution was removed, images were captured by using a confocal laser scanning microscope (CLSM). BMSCs with a density of 5×10^3^ were seeded into the hydrogels for 24 h, and the medium was removed. All the samples were immobilized by 4% paraformaldehyde for 30 min, permeabilized in 0.5% Triton X-100 for 15 min, and blocked in 1% BSA solution for 60 min. Then, to observe the cell adhesion on the hydrogels, these samples were stained with FITC-phalloidin and DAPI, and the images were obtained with a CLSM.

### 2.6 *In vitro* angiogenesis assay

To assess the anti-angiogenic effect of Bevacizumab, the tube formation assay was performed: 300 μL of Matrigel was added to each well in a 24-well Transwell plate and incubated at 37°C with 5% CO_2_ for 4 h, and 100 μL of a suspension [(2×10^5^)/mL] of human umbilical vein endothelial cells (HUVECs) was added to each well; this was followed by placing the Transwell chambers in the culture plate and incubating for 24 h. The hydrogels of each group were placed in the Transwell plate in the upper chamber of the culture plate. After 24 h of culture, random images were collected to observe the tubule formation, and the number of tubular branches was determined using the Image J software. Moreover, cells were collected and the total RNA was extracted using an RNA extraction kit. Then, RT-PCR was performed to detect the expression level of genes specific to angiogenesis, such as VEGF and HIF-1α. The endogenous control gene was GAPDH. The relative gene expression was measured by the 2^−ΔΔCT^ method.

### 2.7 *In vitro* chondrogenic differentiation

To assess the chondrogenic differentiating effect of IGF-1, BMSCs were inoculated with a density of 2×10^4^ in a 24-well Transwell plate. After 24 h of culture, the hydrogel of each group was placed in the upper chamber of the Transwell plate. Then, after 14 and 21 days of culture, cells were collected and the total RNA was extracted using an RNA extraction kit. Then, Reverse Transcription-polymerase Chain Reaction (RT-PCR) was performed to detect the expression levels of osteogenic genes—such as Runx2, Col1a1, and OPN—and chondrogenic genes—such as Col2a1, ACAN, and Sox-9—using GAPDH as an internal reference gene. The relative gene expression was measured by the 2^−ΔΔCT^ method.

### 2.8 *In vivo* growth plate regeneration

Animal experiments were approved by the Ethics Committee of Nanjing Medical University. All experimental procedures on animals were carried out in accordance with the National Institutes of Health guide for the care and use of Laboratory animals. We purchased 40 6-week-old male New Zealand white rabbits, from Nanjing Medical University and randomly divided them into five groups, which included control, GP, GPB, GPI, and GPIB groups.

To study the role of hydrogels in the repair of growth plate injuries, referring to the modeling method of previous studies (Erickson et al., 2017), the proximal tibial growth plate injury model was established. After anesthetizing the rabbits, the surgical area from the medial malleolus to the pelvis was culled clean of fur and then sterilized with gauze soaked in povidone-iodine. A skin incision of about 2 cm was made from the medial gap of the knee joint toward the proximal tibia, and the soft tissue was separated to expose the growth plate of proximal tibia (Supplementary Figure S1A). An incision of approximately 0.5 cm was made from the growth plate to the lower end of the skin incision using a scalpel at the proximal tibia, and the surrounding fascia and soft tissue were scraped off. A 3-mm-diameter drill was used to drill a cortical window in the tibial stem at 10,000 rpm (Supplementary Figure S1B). Then, the drill was placed at an appropriate angle and oriented such that it penetrated through the cortical window up to the center of the growth plate (Supplementary Figure S1C). The appropriate depth of injury was ensured by measuring the entry length of the drill. After ensuring the injury to the growth plate, the drill channel was flushed with sterile saline through a syringe. Thus, the model was successfully established. Next, the hydrogel of each group was injected into the defect, and after closure with bone wax, the subcutaneous tissue was continuously stitched layer by layer, and the skin was sutured. After surgery, the rabbits were kept in separate cages without immobilizing the limbs. Penicillin was intramuscularly injected daily at 20,000 units/kg for 3 days postoperatively to reduce the risk of infection. At 1 month after the surgery, half of the rabbits in each group were euthanized, while the rest were euthanized after 3 months. The histological sections of the lower limb were analyzed through HE staining. Additionally, the repair and regeneration of growth plate cartilage were evaluated by methods of Toluidine blue and Alcian blue staining.

### 2.9 Statistical analysis

The experimental data were expressed as mean ± standard deviation (Mean ± SD). The statistical analysis was performed using GraphPad Prism 8.0. Multi-group comparisons were analyzed by oneway analysis of variance (ANOVA) with the Tukey test. All statistical tests were evaluated as two-sided, and signifificance was set at *p* < 0.05.

## 3 Results

### 3.1 Fabrication and characterization of microspheres

**FIGURE 2:**
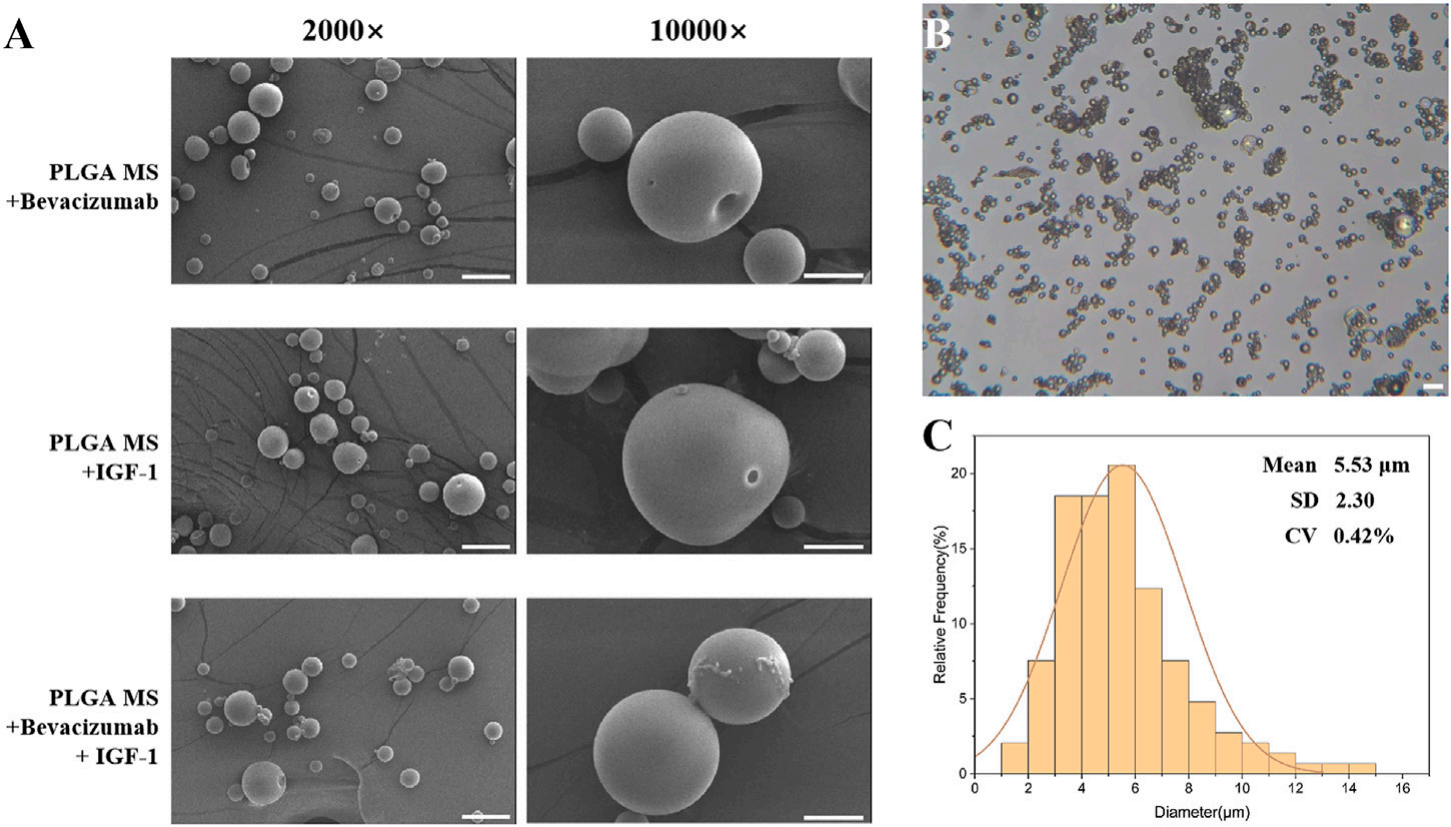
Characteristics of poly (lactic-co-glycolic acid) (PLGA) microspheres. **(A)** Scanning electron micrographs of PLGA microspheres; scale bar = 20 μm. **(B,C)** Particle size distribution of PLGA microspheres; scale bar = 20 μm.

In this study, the preparation of single-layer and bilayer poly (lactic-co-glycolic acid) (PLGA) microspheres loaded with Bevacizumab and insulin-like growth factor-1 (IGF-1) was carried out. Scanning electron microscopy (SEM) images revealed that the microspheres were well-formed with spherical shapes and smooth surfaces, without any signs of adhesion between the particles (Figure 2A). Particle size analysis showed that the average diameter of the microspheres was 5.53 ± 2.30 μm, with a low coefficient of variation (CV) of 0.42% (Figures 2B, C). The data showed that the fabricated PLGA microspheres have a 46.14%% encapsulation efficiency of IGF-1 (1,153.4143 ng of IGF-1 per mg of PLGA microspheres). These results indicate the successful preparation of the microspheres.

### 3.2 Fabrication and characterization of composite hydrogels

**FIGURE 3:**
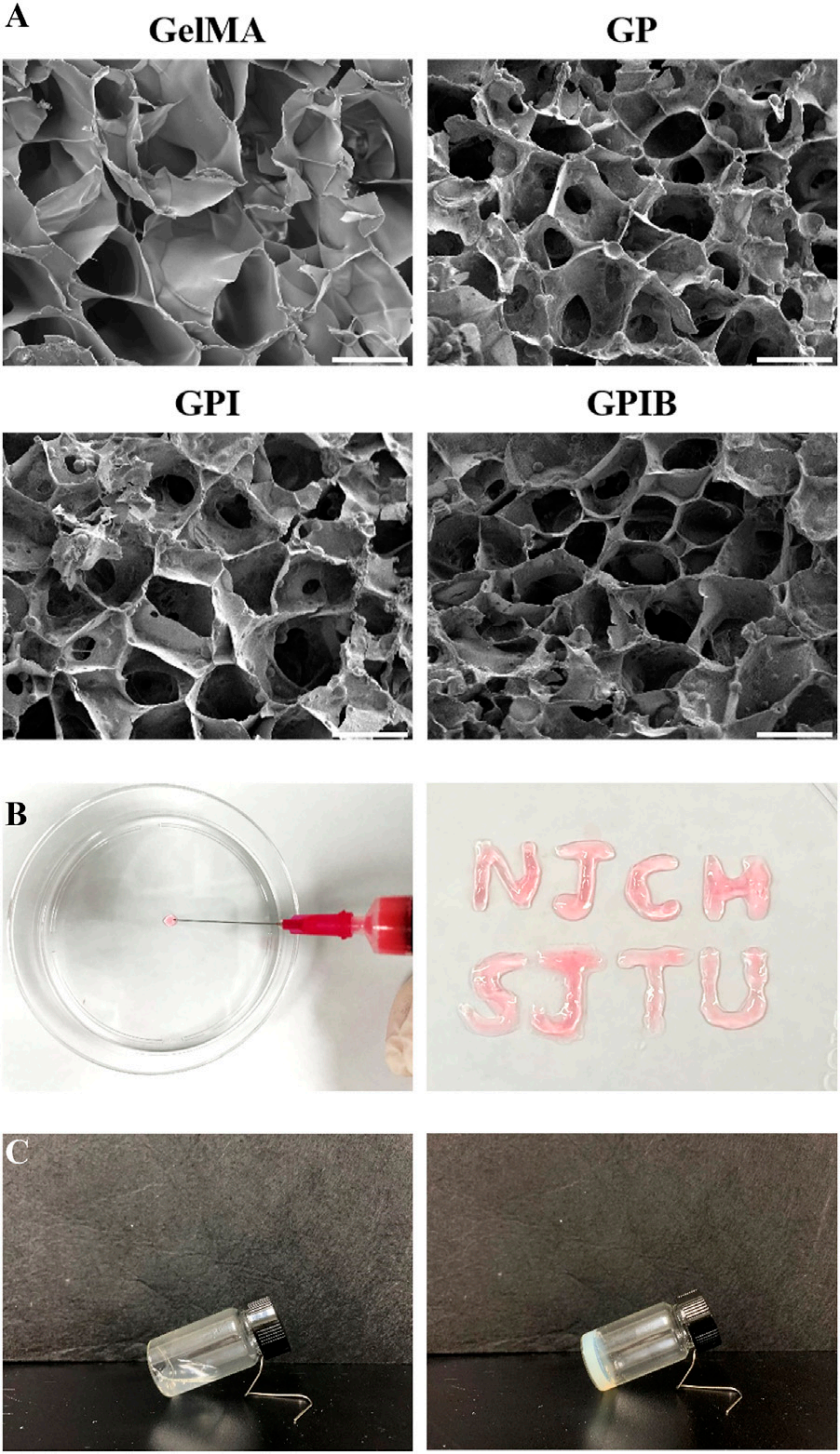
Characteristics of composite hydrogels. **(A)** Scanning electron micrographs of composite hydrogels in each group; scale bar = 50 μm. **(B)** Injectability of composite hydrogels. **(C)** Solidification of composite hydrogels.

**FIGURE 4:**
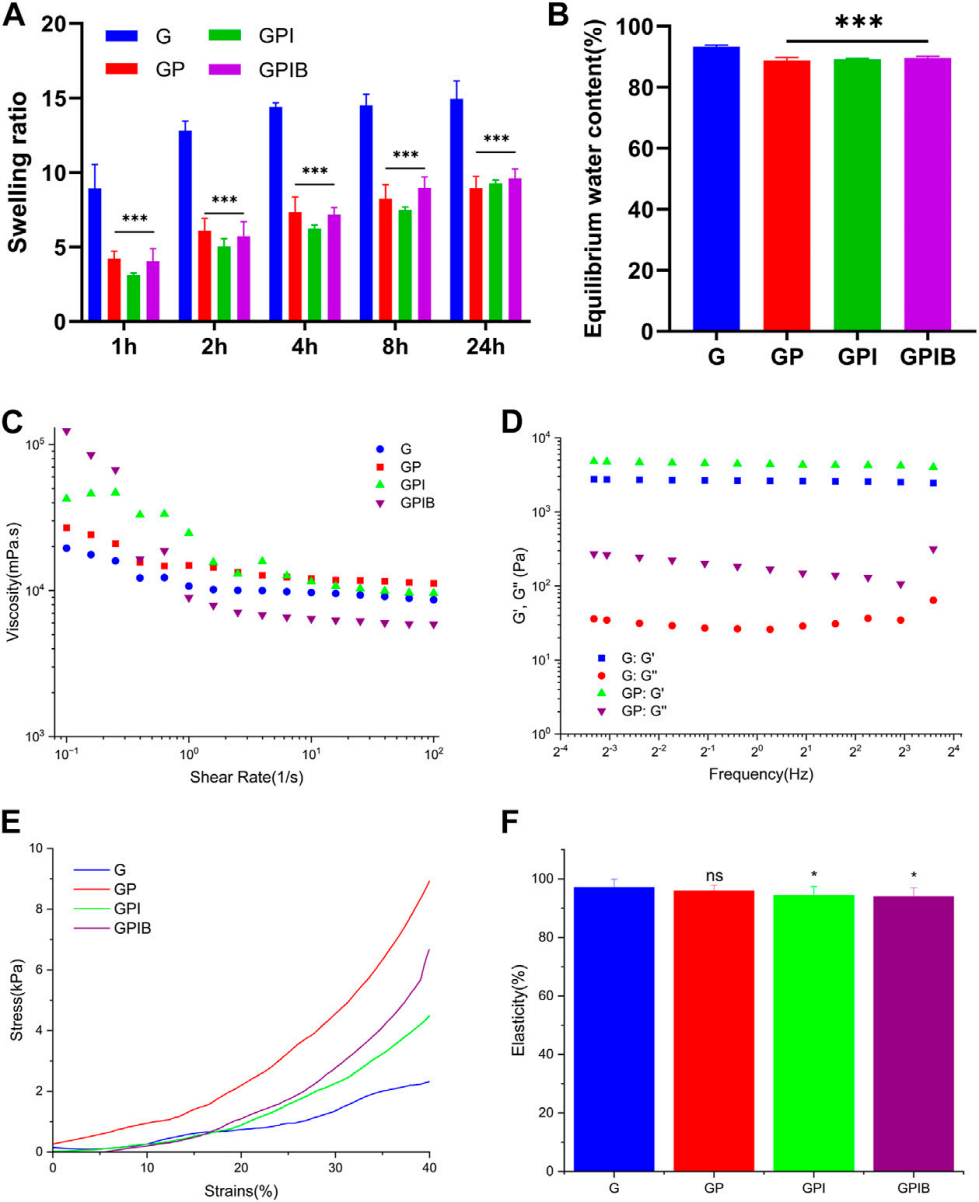
*In vitro* relevant tests of composite hydrogels. **(A)** Swelling ratio and **(B)** equilibrium water content of composite hydrogels in each group. **(C)** Viscosity and shear rate of prepolymer solution in each group. **(D)** Storage modulus (G′) and loss modulus (G″) of composite hydrogels in each group. **(E)** The stress-strain curve and **(F)** elasticity of composite hydrogels in each group. *, *p* < 0.05; **, *p* < 0.01; ***, *p* < 0.001; ns, no significance.

The composite hydrogels were prepared by incorporating the PLGA microspheres of each group into GelMA containing BMSCs. The injectability of the hydrogel was demonstrated in Figure 3B, where the composite hydrogel prepolymer solution was successfully injected using a syringe, followed by cross-linking with ultraviolet (UV) light irradiation. The shear thinning evaluation was conducted, with the results displayed in Figure 4C, which indicated that all the hydrogels exhibited shear thinning properties, thereby facilitating their injection using a syringe. The appearance of the composite hydrogel changed from translucent to opaque and milky white after cross-linking, as observed during the glass bottle tilting test, with a solidification time of 30 s (Figure 3C). The cross-sectional images of the hydrogels in all groups revealed similar sponge-like structures with porous morphologies (Figure 3A). This indicated that the incorporation of PLGA microspheres did not alter the original porous architecture of the GelMA hydrogel. Furthermore, under high magnification, no agglomerated microspheres were observed, showing that the microspheres were well distributed in the hydrogels. This interconnected porous structure could facilitate cell growth, the exchange of nutrients and metabolic wastes, and the construction of a regenerative microenvironment conducive to growth plate cartilage tissue repair.

The swelling properties of the hydrogels play a crucial role in facilitating the efficient exchange of nutrients and metabolic waste within cells. After being immersed in PBS for 24 h, the weight of hydrogels became about 10–15 times that before swelling, and there was a significant decrease in the swelling rate observed in the presence of microspheres (Figure 4A). Additionally, the equilibrium water content (EWC) dropped slightly from 92% to 89% with the addition of microspheres (Figure 4B), indicating an increase in the crosslink density. These results suggest that the GelMA hydrogel and microspheres exhibit a stable crosslinked relationship.

To investigate the effect of microspheres on the mechanical properties of hydrogels, mechanical tests were performed. As illustrated in Figure 4E, the addition of microspheres to GelMA hydrogel improved the mechanical properties of the composite hydrogels. However, as depicted in Figure 4F, the elasticity of composite hydrogels in the GPI group and GPIB group was weaker than that of pure GelMA, yet upon compression, the recovery height of the composite hydrogels was close to the original height, suggesting that the composite hydrogel also basically retained the good elasticity of GelMA. To investigate the effect of the addition of microspheres into the GelMA hydrogel on rheological properties, the strain sweep was conducted to measure the G′ and G″ data. Results showed that hydrogels of all groups had a relatively stable G′ and G″, signifying stable crosslinking. G′ was higher than G″, indicating rapid gelation of the composite hydrogels after crosslinking (Figure 4D).

### 3.3 *In vitro* release behaviour

**FIGURE 5:**
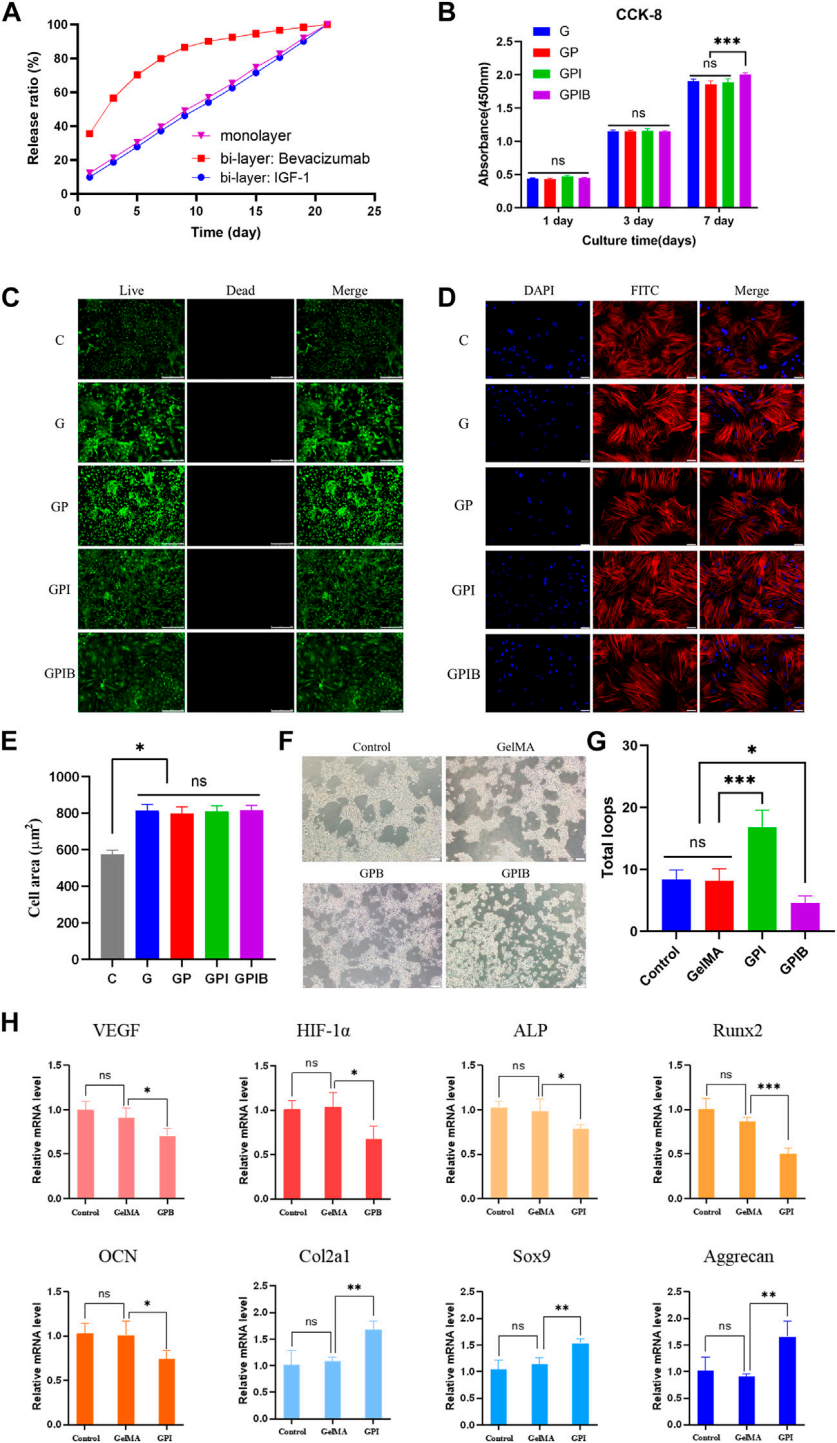
Effects of composite hydrogels on cell viability, morphology, and behavior. **(A)** *In vitro* drug release curve of composite hydrogels. **(B)** Cell proliferation of bone marrow mesenchymal stem cells (BMSCs) cultured on composite hydrogels in each group after culturing for 1, 3, and 7 days. **(C)** Live/Dead staining of BMSCs on composite hydrogels in each group after 24 h of culture; scale bar = 250 μm. **(D)** Confocal laser scanning microscope images of BMSCs cultured on composite hydrogels in each group after 24 h staining with FITC-phalloidin and DAPI; scale bar = 50 μm. **(E)** Quantitative analysis of the cell spreading area. **(F)** Tube formation assay images of HUVECs treated with PBS or composite hydrogels in each group; scale bar = 200 μm. **(G)** The number of total loops in HUVECs treated with PBS or composite hydrogels in each group; **(H)** RT-PCR analysis of angiogenesis-related genes (VEGF and HIF-1α) in HUVECs, and osteogenesis-related genes (Runx2, Col1a1, and OPN) and chondrogenesis-related genes (Col2a1, Sox9, and Aggrecan) in BMSCs. *, *p* < 0.05; **, *p* < 0.01; ***, *p* < 0.001; ns, no significance.

The *in vitro* drug release behavior of hydrogels loaded with PLGA microspheres was analyzed. The results showed that the drug release pattern of hydrogels containing monolayer PLGA microspheres was consistent with a slow and sustained release profile, with no sudden burst effect observed within the first 24 h. On the other hand, the hydrogels loaded with bilayer microspheres exhibited a sequential release pattern, with approximately 80% of Bevacizumab being released in the first week, while only a lesser amount of IGF-1 was released. Over the following 2 weeks, the release of IGF-1 increased steadily and the cumulative release of IGF-1 was higher than that of Bevacizumab (Figure 5A). These findings suggest that the drug release pattern of bilayer microspheres differs from that of monolayer microspheres.

### 3.4 Biocompatibility of composite hydrogels *in vitro*

The hydrogels of each group were co-cultured with BMSCs, and their effects on the proliferation of BMSCs were determined using the CCK-8 assay. The results showed that the cell proliferation activity of each experimental group was slightly lower than that of the control group on days 1, 4, and 7, however, the differences between each experimental group and the control group were not statistically significant (Figure 5B). In addition, to assess the biocompatibility of hydrogels in each group, Live/Dead staining was performed on BMSCs after 24 h of culture. Results showed that the cell survival rate within the hydrogels of each group was high (Figure 5C), indicating that the hydrogels had no cytotoxicity and displayed good biocompatibility.

After 24 h of culture, FITC-phalloidin and DAPI were utilized to label the nucleus and actin cytoskeleton, and then, the morphology of BMSCs in the hydrogels was visualized using confocal laser scanning microscope (CLSM). The results indicated that BMSCs were distributed within the hydrogels and exhibited good attachment (Figure 5D). Additionally, the BMSCs cultured on the surface of the GelMA hydrogel displayed the spindle-like shape, while those on the surface of the composite hydrogels were polygonal. The change in cellular morphology indicated that the addition of microspheres enhanced cell adhesion. As shown in Figure 5E, the spreading area of the BMSCs in all experimental groups was larger than that of the blank group, and the difference was statistically significant (*p* < 0.05). This could be attributed to the fact that the addition of microspheres resulted in a rougher morphology of the composite hydrogel, which facilitated cellular spreading. However, there was no statistically significant difference in the cellular spreading area among the experimental groups.

### 3.5 *In vitro* angiogenic and chondrogenic evaluation

As for tube formation assay *in vitro*, vessels formed by HUVECs loaded on Matrigel and cultured with the hydrogels of each group. IGF-1 is well-established to have an angiogenic effect, and the outcomes illustrated that HUVECs cultured with GPI hydrogels generated dense and complete tubule-like networks, which were more prominent than those produced by the control and GelMA groups. Conversely, the tubule network formed in the GPIB group exhibited sparsity and local fracturing (Figure 5F). And in terms of number of loops, the GPI group demonstrated significantly higher values in comparison to the control and GelMA groups, whereas the number of loops in the GPIB group was significantly lower (Figure 5G). Thus, it is reasonable to conclude that hydrogels of the GPIB group may have the ability to inhibit the tube formation of HUVECs.

Furthermore, to determine the expression of vasculogenic, osteogenic, and chondrogenic genes, RT-PCR was performed, which revealed that the expression of VEGF and HIF-1α was significantly lower (*p* < 0.05) in the GPB group and the bilayer microsphere group compared to the control and GP groups (Figure 5H). This indicates that Bevacizumab could suppress the expression of VEGF. Additionally, RT-PCR data revealed a decrease in osteogenic gene expression (*p* < 0.05) and an increase in chondrogenic gene expression (*p* < 0.05) in the GPI groups (Figure 5H).

### 3.6 *In vivo* growth plate regeneration performance

**FIGURE 6:**
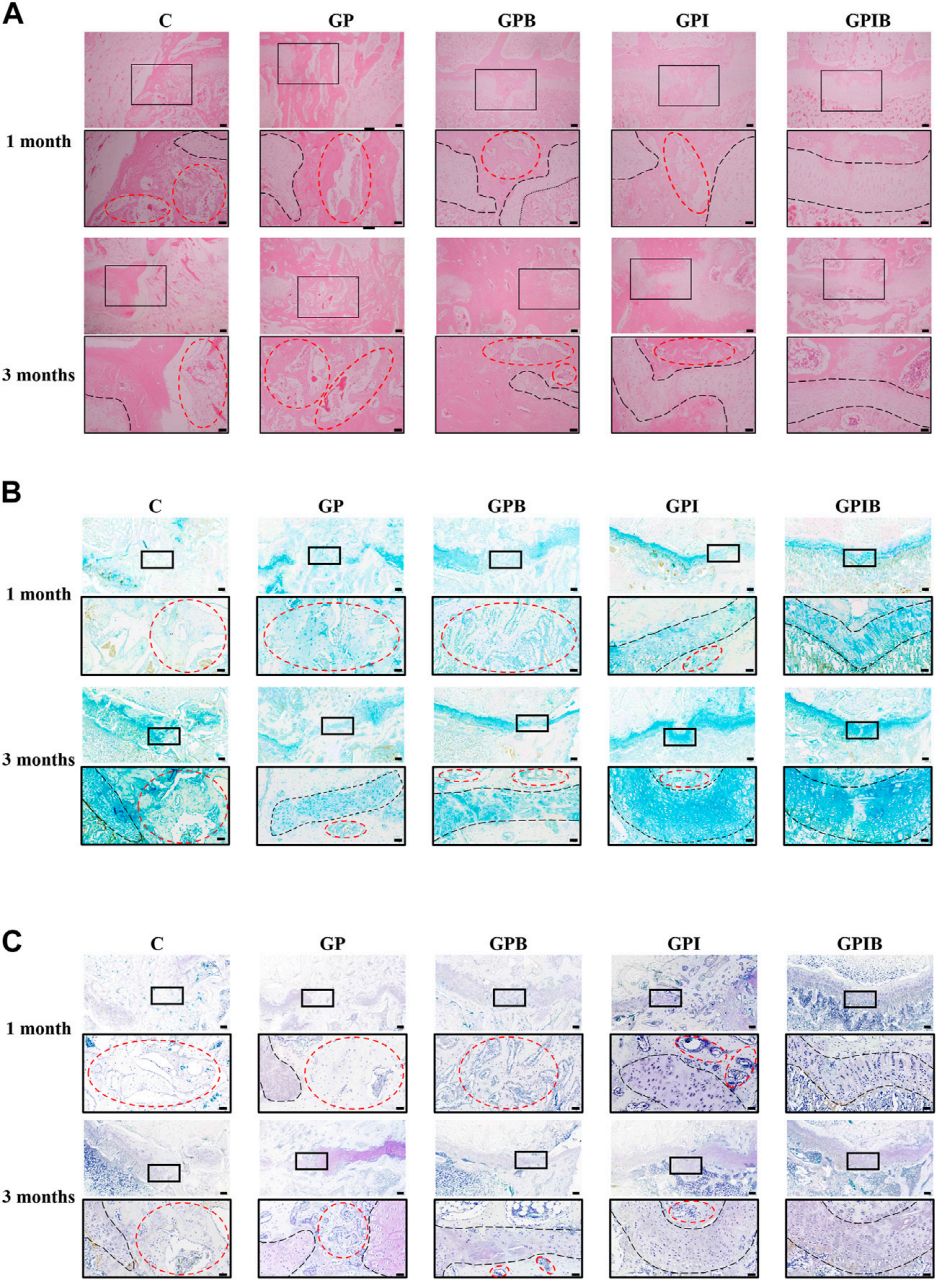
Repair effect of composite hydrogels on growth plate after injury. The HE **(A)**, AB **(B)** and TB **(C)** staining images of the injury site in the growth plate (scale bar = 200 μm). The boxed area represents the defect site and is enlarged below it (scale bar = 50 μm). The region marked by red dotted lines mainly represents bone tissue, and the region marked by black dotted lines mainly represents the growth plate cartilage.

At 1 month and 3 months postoperatively, the specimens were removed and histologically sectioned and stained (Supplementary Figure S2). Histological staining in each group showed a significant formation of cartilage regeneration within the defect area in the GPI and GPIB groups compared to the control group (Figure 6A). Specifically, in the control and GP groups, bone bridge formation was significant and cartilage regeneration was limited at the injury site when compared to the uninjured site. And in the GPB group, bone bridge formation was less compared to the control and GP groups, while cartilage regeneration was still limited, indicating that although the extent of bone bridge formation was reduced, the regeneration of cartilage tissue remained insufficient. In the GPI group, cartilage regeneration was greater compared to the control and GP groups, but the regenerated cartilage displayed a discontinuous or irregular morphology, which may be attributed to the presence of bone tissue that limited the space for cartilage regeneration. Finally, in the GPIB group, prominent cartilage regeneration was observed with minimal bone tissue formation, and the regenerated cartilage exhibited a continuous and regular morphology that closely resembled that of uninjured growth plate cartilage.

Similar to the results of HE staining, AB and TB staining also showed that the control and GP groups had little cartilage formation and more bone and mesenchymal tissue infiltration in the injury site. The bone tissue formation was reduced in the GPB group compared to the control group, but regenerated cartilage was still less and appeared atypical. The GPI group showed typical cartilage regeneration, but the regenerated cartilage appeared intermittent or irregular, which was mainly due to bone bridge formation affecting the regeneration of cartilage. In contrast, the regenerated cartilage tissue in the GPIB group was more continuous, flatter, and more regular, much like the native growth plate cartilage (Figures 6B, C).

## 4 Discussion

In previous studies in which tissue engineering techniques were used for the repair and regeneration of growth plate cartilage, the challenges of the formation of large bone bridges and low chondrogenic efficiency remained (Sundararaj et al., 2015; Li et al., 2017; Erickson et al., 2021a). Through the analysis of the pathological processes following growth plate injury, we posited that the traditional focus on either promoting chondrogenic differentiation and cartilage regeneration or inhibiting the formation of bone bridges may be the underlying reason for the limited success in these efforts. As such, we proposed a novel approach, where the inhibition of osteogenic differentiation and bone bridge formation would be prioritized before promoting chondrogenic differentiation. The sequential and synergistic integration of these two processes was hypothesized to lead to more effective repair and regeneration of cartilage tissue following growth plate injury.

In this study, we aimed to address the aforementioned challenges associated with the repair and regeneration of growth plate cartilage using tissue engineering techniques. To this end, we proposed to first inhibit osteogenic differentiation and bone bridge formation, and then promote chondrogenic differentiation. However, the conventional single layer PLGA microspheres could not provide the desired sequential release of the drugs. Thus, a bilayer drug-loaded PLGA microsphere was adopted in this study, which allowed for sequential release of Bevacizumab and IGF-1. The results of the *in vitro* study showed that the bilayer microspheres were able to release Bevacizumab and IGF-1 in a sequential manner. In particular, the dominant release of Bevacizumab from the outer layer in the first week. Since the fibrogenic phase occurs mainly in the 1st week after growth plate injury, the dominant release of Bevacizumab from the bilayer microspheres in the 1st week can precisely and rapidly inhibit the local neovascularization to the maximum extent and achieve the inhibition of bone bridge formation at the source. Subsequently, the gradual increase in the release of IGF-1 from the inner layer continued to promote the differentiation of the stem cells involved in repair into cartilage, leading to cartilage tissue regeneration.

Furthermore, the composite hydrogel formed from the bilayer microspheres and BMSCs in GelMA showed good injectability and biocompatibility, and was able to effectively block pathological changes associated with bone bridge formation and induce differentiation of stem cells towards chondrocytes, thereby directly and indirectly promoting the regenerative repair of growth plate cartilage. These findings demonstrate the potential of the bilayer drug-loaded PLGA microspheres for the repair and regeneration of growth plate cartilage after injury.

The results of *in vitro* experiments demonstrated that Bevacizumab effectively inhibits angiogenesis, and IGF-1 induces chondrogenic differentiation of stem cells. After a growth plate injury, endogenous stem cells migrate to the injury site and are subjected to a microenvironment that is detrimental to cartilage regeneration, as a result of the inflammatory response and vascular invasion. The majority of the stem cells involved in tissue repair differentiate into osteoblasts under the influence of osteogenic factors, reducing the pool of stem cells available for chondrogenic differentiation and leading to a decrease in the amount of regenerated cartilage tissue. Hence, inhibiting bone bridge formation can indirectly enhance cartilage regeneration. The *in vivo* animal experiments showed that the bilayer microsphere group exhibited superior growth plate cartilage repair and a reduced extent of bone bridge formation due to the early-stage inhibition of angiogenesis (Wang et al., 2011; Chung et al., 2014). Additionally, IGF-1 facilitated the differentiation of stem cells into chondrogenic cells during the repair process, further augmenting the efficacy of cartilage repair (Moon et al., 2015; Sundararaj et al., 2015).

To sum up, the present study proposes a novel bilayer PLGA microsphere-based composite hydrogel for the repair and regeneration of growth plate cartilage following injury. The outer layer of the PLGA microspheres is loaded with Bevacizumab, which effectively inhibits angiogenesis, while the inner layer is loaded with IGF-1, which promotes chondrogenic differentiation of stem cells. By combining the bilayer PLGA microspheres with GelMA, a composite hydrogel was prepared that enables sequential release of the drugs and provides good injectability and biocompatibility. The results of both *in vitro* and *in vivo* experiments demonstrated that the composite hydrogel effectively inhibited bone bridge formation and promoted cartilage regeneration, thereby providing a promising strategy for the treatment of growth plate cartilage injuries.

## Data Availability

The original contributions presented in the study are included in the article/Supplementary Material, further inquiries can be directed to the corresponding author.

## References

[B1] Agirdil Y. (2020). The Growth Plate: A physiologic overview. EFORT Open Rev. 5 (8), 498–507. 10.1302/2058-5241.5.190088 32953135 PMC7484711

[B2] Azarpira M. R. Shahcheraghi G. H. Ayatollahi M. Geramizadeh B. (2015). Tissue engineering strategy using mesenchymal stem cell-based chitosan scafolds in Growth Plate surgery: A preliminary study in rabbits. Orthop. Traumatol. Surg. Res. 101 (5), 601–605. 10.1016/j.otsr.2015.04.010 26188876

[B3] Cepela D. J. Tartaglione J. P. Dooley T. P. Patel P. N. (2016). Classifications in brief: Salter-harris classification of pediatric physeal fractures. Clin. Orthop. Relat. Res. 474 (11), 2531–2537. 10.1007/s11999-016-4891-3 27206505 PMC5052189

[B4] Chase J. L. (2008). Clinical use of anti-vascular endothelial growth factor monoclonal antibodies in metastatic colorectal cancer. Pharmacotherapy 28, 23S–30S. 10.1592/phco.28.11-supp.23S 18980549

[B5] Chen K. Liao S. Li Y. Jiang H. Liu Y. Wang C. (2021). Osteoblast-derived Egfl6 couples angiogenesis to osteogenesis during bone repair. Theranostics 11 (20), 9738–9751. 10.7150/thno.60902 34815781 PMC8581413

[B6] Chen L. Huang X. Chen H. Bao D. Su X. Wei L. (2023). Hypoxia-mimicking scaffolds with controlled release of DMOG and PTHrP to promote cartilage regeneration via the HIF-1α/YAP signaling pathway. Int. J. Biol. Macromol. 226, 716–729. 10.1016/j.ijbiomac.2022.12.094 36526060

[B7] Cho H. Kim J. Kim S. Jung Y. C. Wang Y. Kang B. J. (2020). Dual delivery of stem cells and insulin-like growth factor-1 in coacervate-embedded composite hydrogels for enhanced cartilage regeneration in osteochondral defects. J. Control Release 327, 284–295. 10.1016/j.jconrel.2020.08.002 32763434

[B8] Chung R. Xian C. J. (2014). Recent research on the Growth Plate: Mechanisms for Growth Plate injury repair and potential cell-based therapies for regeneration. J. Mol. Endocrinol. 53 (1), T45–T61. 10.1530/JME-14-0062 25114207

[B9] Chung R. Foster B. K. Zannettino A. C. Xian C. J. (2009). Potential roles of growth factor pdgf-bb in the bony repair of injured Growth Plate. Bone 44 (5), 878–885. 10.1016/j.bone.2009.01.377 19442606

[B10] Chung R. Foster B. K. Xian C. J. (2011). Injury responses and repair mechanisms of the injured Growth Plate. Front. Biosci. Sch. Ed. 3 (1), 117–125. 10.2741/s137 21196362

[B11] Chung R. Foster B. K. Xian C. J. (2014). The potential role of vegf-induced vascularisation in the bony repair of injured Growth Plate cartilage. J. Endocrinol. 221 (1), 63–75. 10.1530/JOE-13-0539 24464023

[B12] Erickson C. B. Shaw N. Hadley-Miller N. Riederer M. S. Krebs M. D. Payne K. A. (2017). A rat tibial Growth Plate injury model to characterize repair mechanisms and evaluate Growth Plate regeneration strategies. J. Vis. Exp. (125), 55571. 10.3791/55571 28715376 PMC5608538

[B13] Erickson C. Stager M. Riederer M. Payne K. A. Krebs M. (2021). Emulsion-free chitosan-genipin microgels for Growth Plate cartilage regeneration. J. Biomater. Appl. 36 (2), 289–296. 10.1177/0885328221999894 33709832 PMC8319035

[B14] Erickson C. B. Newsom J. P. Fletcher N. A. Yu Y. Rodriguez-Fontan F. Weatherford S. A. (2021). Anti-vegf antibody delivered locally reduces bony bar formation following physeal injury in rats. J. Orthop. Res. 39 (8), 1658–1668. 10.1002/jor.24907 33179297

[B15] Eswaramoorthy R. Chang C. C. Wu S. C. Wang G. J. Chang J. K. Ho M. L. (2012). Sustained release of pth(1-34) from plga microspheres suppresses osteoarthritis progression in rats. Acta Biomater. 8 (6), 2254–2262. 10.1016/j.actbio.2012.03.015 22414620

[B16] Foster B. K. John B. Hasler C. (2000). Free fat interpositional graft in acute physeal injuries: The anticipatory langenskiold procedure. J. Pediatr. Orthop. 20 (3), 282–285. 10.1097/01241398-200005000-00002 10823590

[B17] Hasler C. C. Foster B. K. (2002). Secondary tethers after physeal bar resection: A common source of failure? Clin. Orthop. Relat. Res. 405 (405), 242–249. 10.1097/00003086-200212000-00031 12461380

[B18] Hu K. Olsen B. R. (2016). Osteoblast-derived vegf regulates osteoblast differentiation and bone formation during bone repair. J. Clin. Invest. 126 (2), 509–526. 10.1172/JCI82585 26731472 PMC4731163

[B19] Kronenberg H. M. (2003). Developmental regulation of the Growth Plate. Nature 423 (6937), 332–336. 10.1038/nature01657 12748651

[B20] Li W. Xu R. Huang J. Bao X. Zhao B. (2017). Treatment of rabbit Growth Plate injuries with oriented ecm scaffold and autologous bmscs. Sci. Rep. 7, 44140. 10.1038/srep44140 28266598 PMC5339788

[B21] Lui J. C. Colbert M. Cheung C. S. F. Ad M. Lee A. Zhu Z. (2019). Cartilage-targeted igf-1 treatment to promote longitudinal bone growth. Mol. Ther. 27 (3), 673–680. 10.1016/j.ymthe.2019.01.017 30765323 PMC6404097

[B22] McCarty R. C. Xian C. J. Gronthos S. Zannettino A. C. Foster B. K. (2010). Application of autologous bone marrow derived mesenchymal stem cells to an ovine model of Growth Plate cartilage injury. Open Orthop. J. 4, 204–210. 10.2174/1874325001004010204 20721323 PMC2923344

[B23] Moon P. D. Kim M. H. Oh H. A. Nam S. Y. Han N. R. Jeong H. J. (2015). Cysteine induces longitudinal bone growth in mice by upregulating igf-I. Int. J. Mol. Med. 36 (2), 571–576. 10.3892/ijmm.2015.2257 26101100

[B24] Morille M. Toupet K. Montero-Menei C. N. Jorgensen C. Noel D. (2016). Plga-based microcarriers induce mesenchymal stem cell chondrogenesis and stimulate cartilage repair in osteoarthritis. Biomaterials 88, 60–69. 10.1016/j.biomaterials.2016.02.022 26945456

[B25] Provot S. Schipani E. (2007). Fetal Growth Plate: A developmental model of cellular adaptation to hypoxia. Ann. N. Y. Acad. Sci. 1117, 26–39. 10.1196/annals.1402.076 18056035

[B26] Qiu P. Li M. Chen K. Fang B. Chen P. Tang Z. (2020). Periosteal matrix-derived hydrogel promotes bone repair through an early immune regulation coupled with enhanced angio- and osteogenesis. Biomaterials 227, 119552. 10.1016/j.biomaterials.2019.119552 31670079

[B27] Shaw N. Erickson C. Bryant S. J. Ferguson V. L. Krebs M. D. Hadley-Miller N. (2018). Regenerative medicine approaches for the treatment of pediatric physeal injuries. Tissue Eng. Part B Rev. 24 (2), 85–97. 10.1089/ten.TEB.2017.0274 28830302 PMC5905866

[B28] Shukrimi A. B. Afizah M. H. Schmitt J. F. Hui J. H. (2013). Mesenchymal stem cell therapy for injured Growth Plate. Front. Biosci. Sch. Ed. 5 (2), 774–785. 10.2741/s407 23277086

[B29] Su Y. Zhang B. Sun R. Liu W. Zhu Q. Zhang X. (2021). Plga-based biodegradable microspheres in drug delivery: Recent advances in research and application. Drug Deliv. 28 (1), 1397–1418. 10.1080/10717544.2021.1938756 34184949 PMC8248937

[B30] Sundararaj S. K. Cieply R. D. Gupta G. Milbrandt T. A. Puleo D. A. (2015). Treatment of Growth Plate injury using igf-I-loaded plga scaffolds. J. Tissue Eng. Regen. Med. 9 (12), E202–E209. 10.1002/term.1670 23239617

[B31] Verma V. Verma P. Kar S. Ray P. Ray A. R. (2007). Fabrication of agar-gelatin hybrid scaffolds using a novel entrapment method for *in vitro* tissue engineering applications. Biotechnol. Bioeng. 96 (2), 392–400. 10.1002/bit.21111 16850454

[B32] Villemure I. Stokes I. A. (2009). Growth Plate mechanics and mechanobiology. A survey of present understanding. J. Biomech. 42 (12), 1793–1803. 10.1016/j.jbiomech.2009.05.021 19540500 PMC2739053

[B33] Wang C. J. Huang K. E. Sun Y. C. Yang Y. J. Ko J. Y. Weng L. H. (2011). Vegf modulates angiogenesis and osteogenesis in shockwave-promoted fracture healing in rabbits. J. Surg. Res. 171 (1), 114–119. 10.1016/j.jss.2010.01.045 20452608

[B34] Wang X. Li Z. Wang C. Bai H. Wang Z. Liu Y. (2021). Enlightenment of Growth Plate regeneration based on cartilage repair theory: A review. Front. Bioeng. Biotechnol. 9, 654087. 10.3389/fbioe.2021.654087 34150725 PMC8209549

[B35] Xian C. J. Foster B. K. (2006). Repair of injured articular and Growth Plate cartilage using mesenchymal stem cells and chondrogenic gene therapy. Curr. Stem Cell Res. Ther. 1 (2), 213–229. 10.2174/157488806776956904 18220868

[B36] Xie Y. Zhou S. Chen H. Du X. Chen L. (2014). Recent research on the Growth Plate: Advances in fibroblast growth factor signaling in Growth Plate development and disorders. J. Mol. Endocrinol. 53 (1), T11–T34. 10.1530/JME-14-0012 25114206

[B37] Ye L. Xu J. Mi J. He X. Pan Q. Zheng L. (2021). Biodegradable magnesium combined with distraction osteogenesis synergistically stimulates bone tissue regeneration via cgrp-fak-vegf signaling Axis. Biomaterials 275, 120984. 10.1016/j.biomaterials.2021.120984 34186235

[B38] Zhou F. H. Foster B. K. Sander G. Xian C. J. (2004). Expression of proinflammatory cytokines and growth factors at the injured Growth Plate cartilage in young rats. Bone 35 (6), 1307–1315. 10.1016/j.bone.2004.09.014 15589211

