# Supplementary figures and images for: Injectable hydrogel loaded with bilayer microspheres to inhibit angiogenesis and promote cartilage regeneration for repairing growth plate injury
Source: Front Bioeng Biotechnol. 2023 May 18;11:1181580. doi: 10.3389/fbioe.2023.1181580 (PMC10232875; doi:10.3389/fbioe.2023.1181580)

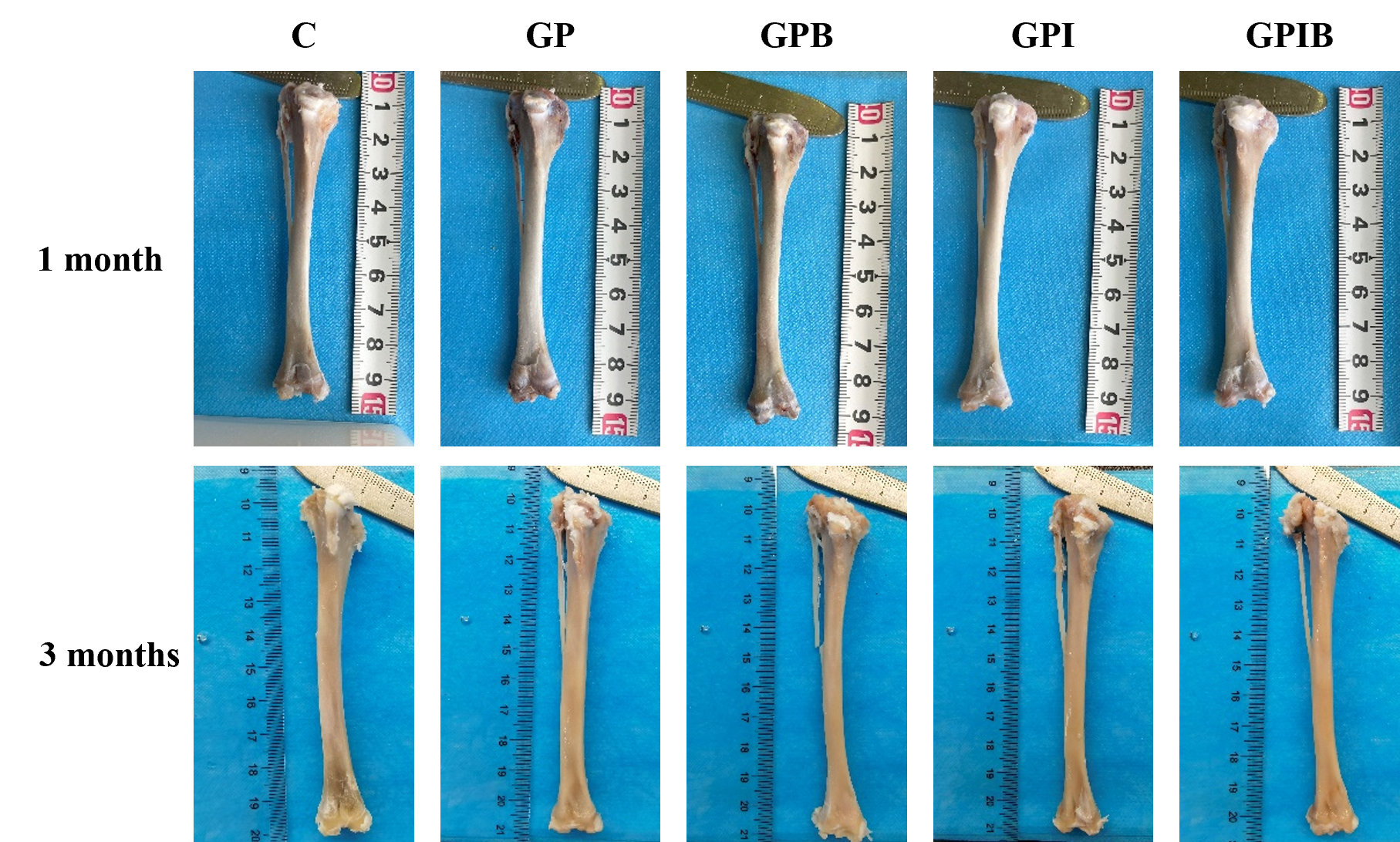

Supplement: Supplementary file 1 [file Image2.TIF]

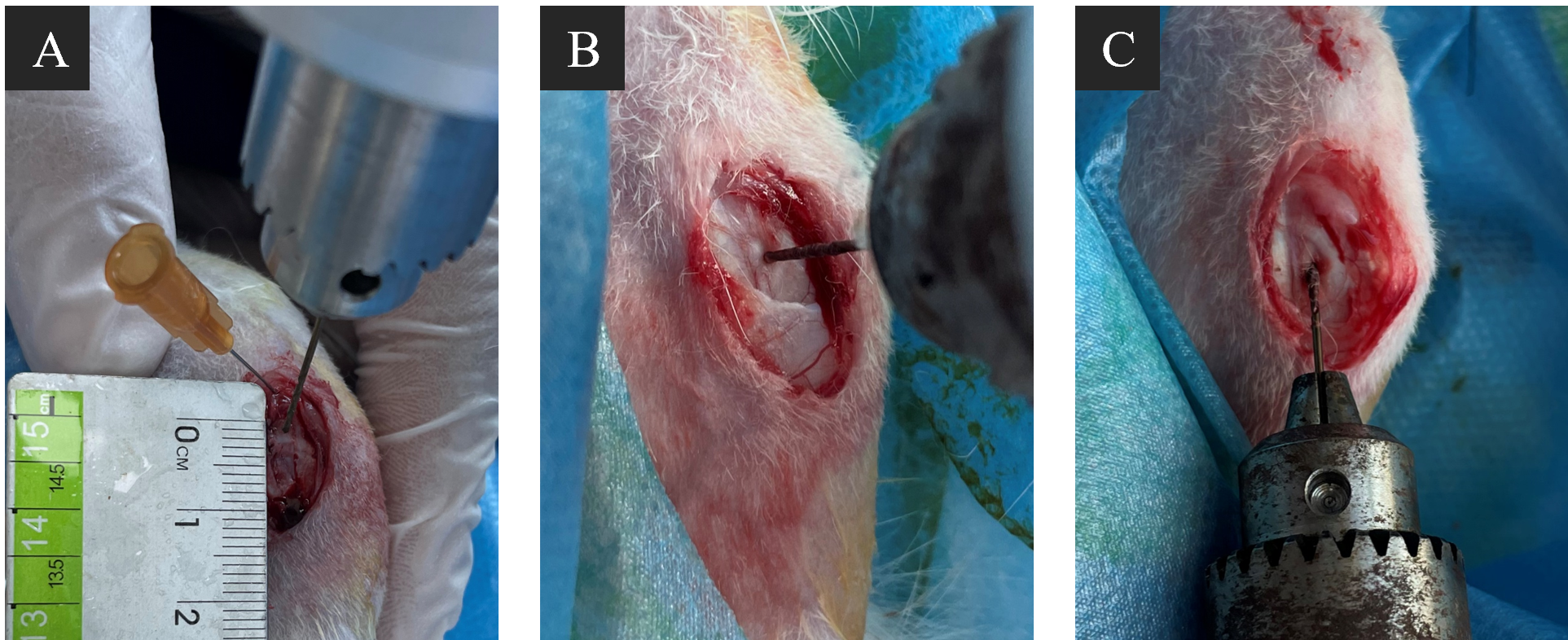

Supplement: Supplementary file 2 [file Image1.TIF]
